# Emerging Role of TRP Channels in Osteoarthritis Pathogenesis

**DOI:** 10.3390/cells15030299

**Published:** 2026-02-05

**Authors:** Shivmurat Yadav, Jyoti Yadav, Mary Beth Humphrey

**Affiliations:** 1 Department of Medicine, College of Medicine, University of Oklahoma Health Campus, Oklahoma City, OK 73104, USA; shivmurat-yadav@ou.edu (S.Y.);; 2 Department of Cell Biology, College of Medicine, University of Oklahoma Health Campus, Oklahoma City, OK 73104, USA; 3 Department of Veterans Affairs, Oklahoma City, OK 73111, USA

**Keywords:** TRP channels, osteoarthritis, synoviocytes, sensory neurons, inflammation, pain

## Abstract

**Highlights:**

**What are the main findings?**

- Transient receptor potential (TRP) channels serve as molecular sensors in chondrocytes, synoviocytes, and sensory neurons in the joint tissues.
- In osteoarthritis, TRP channels play a key role in modulating cartilage homeostasis, synovial inflammation, and pain sensitization through calcium-dependent signaling.

**What are the implications of the main findings?**

- Targeting TRP channels in specific tissues could lead to therapeutics that reduce osteoarthritis pain.
- Targeting TRP channels in specific tissues could lead to therapeutics that promote cartilage health.

**Abstract:**

Osteoarthritis (OA) is a degenerative joint disease characterized by cartilage degradation, synovial inflammation, osteophyte formation, joint space narrowing, and persistent pain. During OA progression, synovial inflammation triggers the release of pro-inflammatory cytokines, including IL-1β, TNF-α, and IL-6, which activate matrix metalloproteinases (MMPs) and aggrecanases, driving extracellular matrix (ECM) degradation. Emerging evidence indicates that transient receptor potential (TRP) channels, via calcium (Ca^2+^) signaling, function as molecular sensors in joint tissues, including chondrocytes, synoviocytes, sensory neurons, and regulate cartilage homeostasis, synovial inflammation, and OA pain. In cartilage, TRP channels govern chondrocyte survival, mechanotransduction, autophagy, oxidative stress, and ECM turnover, thereby modulating cartilage homeostasis. In synovial tissue, TRP channels regulate inflammatory signaling and cytokine, chemokine, and matrix-degrading enzyme production, leading to synovitis and joint destruction. In sensory neurons innervating the joint, TRP channels respond to mechanical and inflammatory stimuli, increasing nociceptor excitability, neuropeptide release, and pain sensitization, driving OA pain. TRP channel signaling also modulates immune cell infiltration and macrophage-driven inflammation, sustaining chronic pain and tissue damage in OA. This review summarizes emerging evidence on TRP channel functions in OA pathogenesis and highlights their potential as therapeutic targets to alleviate inflammation, protect cartilage, and reduce OA-associated pain.

## 1. Introduction

Osteoarthritis (OA) is a multifactorial joint disorder characterized by progressive cartilage deterioration, persistent inflammation, and joint pain. Transient receptor potential (TRP) channels are a diverse family of non-selective cation channels permeable to calcium (Ca^2+^) and other cations that play essential roles in detecting various cellular and environmental stimuli, including mechanical, thermal, chemical, and osmotic stimuli, within joint tissues [1,2]. In mammals, 28 different TRP channel proteins have been identified, grouped into seven subfamilies based on amino acid sequence homology: TRP Ankyrin (TRPA), TRP Canonical (TRPC), TRP Melastatin (TRPM), TRP Mucolipin (TRPML), TRP No-mechano-potential (TRPN), TRP Polycystin (TRPP), and TRP Vanilloid (TRPV) [3]. Articular chondrocytes derived from patients with OA exhibit expression of multiple TRP family ion channels [4]. These channels are essential for regulating sensory perception, cellular signaling, and maintaining physiological balance, acting as key mediators in both normal functions and OA pathophysiology.

**Figure 1:**
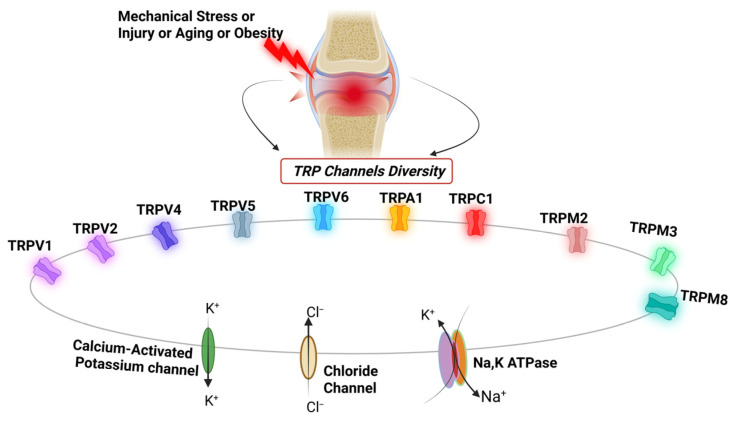
A schematic overview of ion channels known to be involved in osteoarthritis. Various TRP channels, including TRPV1, TRPV2, TRPV4, TRPV5, TRPV6, TRPA1, TRPC1, TRPM2, TRPM3, and TRPM8, regulate various processes in the joint tissue. TRP channel activity is further modulated by membrane potential, which is regulated by potassium and chloride channels, and the Na^+^/K^+^-ATPase.

TRP channels are essential sensors responding to mechanical forces, temperature changes, osmotic stress, inflammatory mediators, and oxidative signals within the joint environment (Figure 1) [2,5]. In OA, these channels are found across several joint tissues, including articular chondrocytes, synovial fibroblasts (SF), osteoblasts, and sensory neurons, where they mediate signals that regulate cartilage health, synovial inflammation, and pain signaling [6,7]. Channels such as TRPV1, TRPA1, TRPV4, and TRPC1 subtypes respond to inflammatory mediators, mechanical overload, oxidative stress, and osmotic changes, all of which are hallmark features of OA [7,8,9,10]. TRP channels, by regulating intracellular Ca^2+^ signaling, play a key role in controlling chondrocyte metabolism, mechanotransduction, and extracellular matrix (ECM) homeostasis, making them crucial regulators of cartilage physiology and pathology. In human and animal SF, some TRP channels, such as TRPV4, are highly expressed [11]. They amplify inflammatory signaling by promoting M1 macrophage polarization and increasing the release of pro-inflammatory cytokines, including IL-1β, IL-6, and TNF-α, thereby leading to tissue damage and inflammation in the OA joint [11]. TRP channels, such as TRPV1 and TRPA1, also play a crucial role in OA-related pain by sensitizing peripheral nociceptors [12]. These channels are highly expressed in sensory neurons that innervate joints and become hypersensitive in OA inflammation, lowering pain thresholds and causing chronic pain. Thus, TRP channels contribute to OA progression by promoting catabolic and inflammatory pathways and driving peripheral nociceptor sensitization.

Ca^2+^ influx through these channels may influence signaling in various cells, including chondrocytes, osteoblasts, osteoclasts, mesenchymal stem cells, and sensory neurons. TRP channels regulate chondrocyte metabolism, apoptosis, and catabolic responses, and serve as vital molecular mediators in the pathogenesis of OA [5,13]. Clinically, pain and inflammation are hallmark features of OA that significantly impact patients’ quality of life; however, the underlying molecular mechanisms of OA pain and inflammation remain inadequately understood. Specific TRP channels, particularly those expressed in dorsal root ganglia (DRG) sensory neurons and joint tissues, are increasingly acknowledged as key regulators of nociception, neurogenic inflammation, and cartilage degeneration [6]. OA progression results from complex interactions among biomechanical stress, inflammation, and cellular dysfunction, involving immune cells and nociceptive sensory nerve cells. TRP channels serve as molecular sensors that translate physical and chemical signals into intracellular Ca^2+^ responses, thereby regulating oxidative stress and preserving the ECM [7,10,14,15].

Comprehending how various TRP channel subtypes contribute to OA-associated pain and tissue remodeling could lead to new approaches for developing mechanism-based therapies that extend beyond mere symptom management to disease modification. By elucidating the connections between mechanical loading, inflammation, and pain pathways, TRP channels represent a pivotal mechanism in OA pathogenesis.

## 2. TRP Channels in Chondrocytes and Cartilage Homeostasis

**Figure 2:**
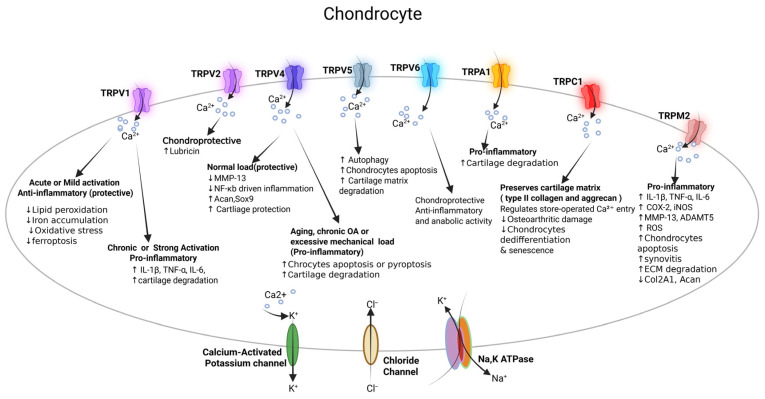
A schematic overview of how TRP channels regulate multiple aspects of osteoarthritis progression in chondrocytes. Normal or acute activation of TRPV1 and TRPV4 leads to anti-inflammatory and cartilage-protective effects, whereas chronic or excessive mechanical stimuli leads to pro-inflammatory and cartilage-damaging effects. TRPV2 exerts chondroprotective effects by enhancing lubricin production and reducing synovitis; TRPV5 is associated with autophagic and apoptotic pathways that contribute to cartilage breakdown; and TRPV6 favors anabolic signaling and dampens inflammatory responses. TRPA1 and TRPM2 are pro-inflammatory and promote cartilage degradation, whereas TRPC1 supports basal Ca^2+^ homeostasis and preserves the chondrocyte phenotype by promoting type II collagen and aggrecan synthesis.

TRP channels, particularly TRPV1, TRPV2, TRPV4, TRPV5, TRPV6, TRPC1, TRPM2, and TRPA1, are highly expressed in articular chondrocytes and are sensitive to mechanical stimuli, temperature variations, osmotic pressure, and oxidative stress (Figure 2) [10,16,17,18,19,20,21]. TRPV1 links oxidative stress, inflammation, and ferroptosis in OA [18,22]. Single-cell RNA sequencing analysis identifies ferroptosis, a specific iron-dependent regulated cell death, with ferroptotic chondrocyte clusters characterized by elevated expression of genes involved in lipid peroxidation and iron metabolism, highlighting ferroptosis as a major factor in chondrocyte death and cartilage degeneration in OA [18].

Among the differentially expressed genes, TRPV1 emerged as an important anti-ferroptotic regulator. TRPV1 mediates Ca^2+^ influx and may help maintain redox balance and mitochondrial function by modulating Ca^2+^-dependent antioxidant pathways. Functional validation demonstrates that TRPV1 activation prevents ferroptosis by reducing oxidative stress, decreasing lipid peroxidation, preventing iron accumulation, and maintaining mitochondrial integrity in OA chondrocytes [23]. Loss or inhibition of TRPV1 increases ferroptosis susceptibility, worsening cartilage matrix degradation and inflammation [23]. These results suggest that TRPV1 protects chondrocytes by reducing ferroptosis-related cell death, thereby preventing cartilage loss and slowing OA progression. Mechanistically, TRPV1 activation induces increased expression of Glutathione Peroxidase 4 (GPX4) and solute carrier family 7 member 11 (SLC7A11), two critical anti-ferroptotic factors, via Ca^2+^ signaling–dependent activation of nuclear factor erythroid 2-related factor 2 (Nrf2), a key antioxidant transcription factor [22]. SLC7A11 functions as a cystine/glutamate antiporter that regulates glutathione synthesis, thereby preserving cellular redox balance and protecting cells against oxidative stress. This process strengthens cellular defenses against lipid reactive oxygen species (ROS) and supports chondrocyte survival under inflammatory stress. Additionally, TRPV1 agonists, such as capsaicin, have shown potential to attenuate OA progression by restoring redox homeostasis and suppressing ferroptosis-driven cartilage damage [24].

TRPV2 is a mechanosensitive ion channel found in superficial zone chondrocytes of healthy articular cartilage (Figure 2). It facilitates Ca^2+^ influx in response to mechanical stimulation, thereby increasing lubricin production, a crucial lubricant that protects cartilage surfaces [21]. TRPV2-deficient mice exhibit lowered lubricin levels, increased articular cartilage surface fibrillation, and abnormal endochondral ossification marked by elevated hypertrophic chondrocyte proteins, including runt-related transcription factor 2 (RUNX2) and Collagen type X alpha 1 chain (COL10aA1) [21]. TRPV2 agonist probenecid restores lubricin levels and suppresses pathological cartilage ossification. Overall, these findings suggest that TRPV2-mediated Ca^2+^ signaling supports cartilage health by enhancing lubricin synthesis and inhibiting chondrocyte hypertrophy and calcification, making TRPV2 a promising target for maintaining healthy cartilage.

**Figure 3:**
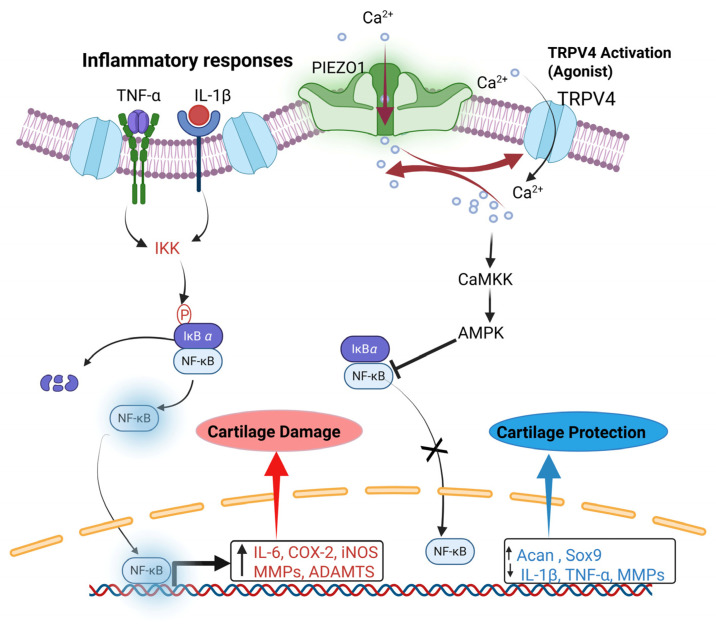
TRPV4 activation enhances anti-inflammatory and anabolic responses in chondrocytes. Pro-inflammatory cytokines activate nuclear factor kappa-light-chain-enhancer of activated B cells (NF-κB) signaling pathways that drive catabolic enzyme expression and cartilage degradation in OA. TRPV4 normal or acute activation, along with PIEZO1 or without PIEZO1, increases Ca^2+^ influx in chondrocytes, which activates AMPK, reduces NF-κB-driven inflammatory signals, and promotes anabolic and cartilage-protective gene expression.

TRPV4 is also expressed in chondrocytes, and studies suggest that it contributes to age-related OA (Figure 2). The TRPV4 channel in chondrocytes facilitates Ca^2+^ influx in response to low-intensity mechanical stimuli, which is essential for physiological mechanotransduction [25]. It works with the piezo-type mechanosensitive ion channel component 1 (PIEZO1), another mechanosensitive channel, to regulate Ca^2+^ oscillations, cytoskeletal tension, and cell adaptation to different mechanical strains (Figure 3) [25]. TRPV4 mainly responds to mechanical stress (3–8% strain), while PIEZO1 responds to stronger or traumatic stress (up to 18%). This complementary signaling allows chondrocytes to adjust to a range of mechanical environments [25]. Unlike PIEZO1, TRPV4 is not directly activated by membrane stretching; instead, it plays an indirect role in mechanotransduction and in maintaining healthy cartilage [25]. Inhibiting TRPV4 can protect against age-related OA but does not prevent trauma-induced OA, suggesting a specific role in normal physiological loading [26].

TRPV4 and PIEZO1 also regulate each other; activating one can reduce the effects of the other (Figure 3). If both are suppressed, cells produce more key ECM proteins, such as aggrecan and type II collagen, promoting cartilage repair [25]. Activation of TRPV4 can influence PIEZO1 channel opening, thereby fine-tuning Ca^2+^ signaling during mechanical adaptation. Pericellular matrix degradation by matrix metallopeptidases (MMPs) disrupts chondrocyte mechanotransduction during OA development [27]. This degradation alters mechanosensitive signaling pathways, mediated by TRPV4 and PIEZO1, reducing Ca^2+^ influx and impairing mechanotransduction [27]. Inhibiting activities of these channels diminishes anabolic responses while increasing catabolic and inflammatory signals in chondrocytes. Consequently, pericellular matrix loss, combined with disrupted TRPV4 and PIEZO1 activity, promotes cartilage degeneration, inflammation, and OA progression, underscoring their critical role in the disease.

TRPV4 also plays a key role in triggering Ca^2+^ influx in response to osmotic changes in primary chondrocytes from mice and pigs, as well as in mouse articular cartilage, which is crucial for mechanotransduction and maintaining cartilage health [26,28,29]. Global and cartilage-specific TRPV4 knockouts reveal contrasting roles of this ion channel in OA. Global *Trpv4* knockout (KO) mice, in which TRPV4 is absent in all tissues from development, disrupt systemic biomechanics and joint physiology, leading to accelerated OA progression [29]. These global *Trpv4* KO mice reveal early, severe, and sex-specific spontaneous OA, initially visible in articular cartilage at 9 months, with males exhibiting more pronounced OA than females [29]. In contrast, cartilage-specific *Trpv4* KO mice show a protective role of TRPV4 against age-related OA; these mice have less cartilage degeneration, reduced subchondral bone sclerosis, and fewer osteophytes than in wild-type mice [28]. These data suggest that TRPV4 promotes cartilage catabolism with aging. Interestingly, the protective effect varied by sex; males experienced greater protection, while females showed milder changes, suggesting hormonal influences on TRPV4 signaling [28]. Therefore, TRPV4 expression in chondrocytes contributes to age-related OA.

Taken together, it appears that the TRPV4 channel plays a complex role in OA, depending on context, which can be both protective and damaging. In healthy cartilage, TRPV4-mediated Ca^2+^ influx supports mechanotransduction, promotes anabolic signaling, ECM synthesis, and maintains cartilage health [26]. It can also be protective by promoting cartilage matrix formation via pathways such as Ca^2+^/calmodulin-dependent protein kinase kinase (CaMKK)/adenosine monophosphate (AMP)-activated protein kinase (AMPK). However, under excessive mechanical stress or inflammation, TRPV4 becomes dysregulated, leading to excess Ca^2+^ entry, mitochondrial dysfunction, oxidative stress, and chondrocyte apoptosis or pyroptosis [30]. Thus, TRPV4 is protective in physiological mechanosensing but pathogenic when overactivated, underscoring the crucial role of balanced activity in cartilage homeostasis in OA.

Other TRP channels, like TRPV5, TRPV6, and TRPC1, have been implicated in Ca^2+^-regulated control of chondrocyte survival (Figure 2). TRPV5 expression is increased in rat cartilage during monosodium iodoacetate (MIA)-induced OA [31]. This increase occurs alongside decreases in chondrocyte autophagy markers, including light chain 3B (LC3B), the LC3-II/LC3-I ratio, and Beclin-1, and elevated levels of calmodulin and B-cell lymphoma 2 (Bcl-2) [31]. LC3B is an autophagy-related protein that undergoes lipidation from LC3-I to LC3-II during autophagosome formation, and the LC3-II/LC3-I ratio is commonly used as a surrogate marker of autophagic activity. TRPV5 inhibitor, ruthenium red, slowed the progression of joint destruction in the MIA model. These results indicate that TRPV5 is upregulated during OA, leading to reduced chondrocyte autophagy and, in turn, promoting chondrocyte apoptosis and cartilage matrix degradation [32].

TRPV6 is essential for regulating chondrocyte function and maintaining cartilage health. It is highly expressed in healthy cartilage but is significantly reduced in osteoarthritic articular cartilage in both rats and humans [17]. In rat OA cartilage, the expression of TRPV1, TRPV4, TRPV5, and collagen type II alpha 1 (Col2a1) decreases along with TRPV6, while MMP-1 and MMP-13 increase [17]. Additionally, TRPV6 mediates Ca^2+^ influx, and its knockdown using siRNA in chondrocytes or its knockout in mice suppresses anabolic gene expression (*Col2a1*, aggrecan [*Acan*]), and increases catabolic enzyme (MMP-1, MMP-13) and pro-inflammatory mediator expression (TNF-α, IL-6) [17]. *Trpv6* KO mice exhibit severe age-related OA features, including cartilage fibrillation, eburnation, and proteoglycan loss [17]. Moreover, the absence of TRPV6 markedly impairs chondrocyte function, resulting in increased inflammation, elevated levels of matrix-degrading enzymes, decreased cell proliferation, and increased chondrocyte apoptosis, highlighting its role in balancing matrix synthesis and degradation [17]. Taken together, TRPV6 acts as a chondroprotective channel, increasing Ca^2+^-dependent anabolic, anti-catabolic, and anti-inflammatory activities. When its function is compromised, Ca^2+^-dependent signaling is disrupted, damaging matrix metabolism and causing chondrocyte dysfunction, which further advances OA progression.

TRPC1, a widely expressed mechanosensitive ion channel, has recently been shown to be protective against OA in a mouse model of destabilized medial meniscus (DMM)- induced OA (Figure 2) [10]. Using human OA cartilage samples and murine post-traumatic OA models, TRPC1-mediated Ca^2+^ signaling protects chondrocytes from stress-induced senescence and supports cartilage integrity [10]. TRPC1 expression is reduced in human OA cartilage compared to non-OA cartilage, and this pattern is observed in mouse cartilage during OA pathogenesis [10]. Functional insights, however, are based mainly on rodent studies. Global *Trpc1* KO mice develop more severe cartilage damage after DMM, as indicated by higher Osteoarthritis Research Society International (OARSI) scores, reduced type II collagen, and higher levels of cartilage breakdown markers, including MMP13 and collagen X, compared with control DMM mice [10]. *Trpc1*-deficient chondrocytes show impaired Ca^2+^ influx in response to thapsigargin, a potent inhibitor of sarco/endoplasmic reticulum calcium-ATPase enzyme that induces endoplasmic reticulum (ER) release of Ca^2+^. *Trpc1*-deficient chondrocytes also exhibit reduced basic fibroblast growth factor (bFGF)- induced responses, accelerated dedifferentiation, and increased expression of cellular senescence markers, including p16 inhibitor of kinase 4a (p16INK4a) and senescence-associated β-galactosidase (SA-β-gal) activity [10]. Transcriptomic analysis reveals enrichment of pathways related to apoptosis, ECM remodeling, and regulation of cell numbers in *Trpc1*-deficient cartilage [10]. While these findings support a protective role for TRPC1 in post-traumatic OA in mice, mechanistic validation in human chondrocytes remains limited. Therefore, conclusions regarding human OA should be interpreted with caution. Additionally, evidence for the roles of TRPV6 and TRPC1 in OA is limited to single studies, underscoring the need for further validation across diverse experimental models and human tissues.

TRPM2 is highly expressed in cartilage from OA patients, post-traumatic OA mice, and inflammatory chondrocytes (Figure 2) [19]. *Trpm2* KO mice are protected against DMM-induced OA progression, while *Trpm2* overexpression results in worsened DMM-induced OA and promotes an OA-like phenotype in chondrocytes [19]. After DMM surgery, *Trpm2*-overexpressing in mice cartilage shows increased levels of catabolic markers, including MMP13 and a disintegrin and metalloproteinase with thrombospondin motifs (ADAMTS)5, and inflammatory markers, including cyclooxygenase-2 (COX-2) and inducible Nitric Oxide Synthase (iNOS), and reduced levels of anabolic markers (COL2A1, ACAN) [19]. Additionally, these *Trpm2*-overexpressing mice also show accelerated osteophyte formation and synovitis consistent with OA. Chelation of Ca^2+^ with BAPTA-AM treatment protected against chondrocyte damage by restoring chondrocyte proliferation, by normalizing abnormal gene expression, and reducing inflammation, apoptosis, and impaired ECM metabolism [19]. Under oxidative stress, TRPM2 activation leads to excessive Ca^2+^ influx and mitochondrial dysfunction, which trigger the cyclic GMP-AMP synthase (cGAS) -stimulator of interferon genes (STING) pathway and subsequent activation of nuclear factor kappa-light-chain-enhancer of activated B cells (NF-κB) [19,33,34]. This cascade promotes the production of pro-inflammatory cytokines, including IL-1β, IL-6, and TNF-α, which increase oxidative stress and further activate TRPM2. This cycle of inflammation, Ca^2+^ overload, and oxidative injury accelerates chondrocyte apoptosis and cartilage degradation [19]. Thus, TRPM2 drives the cartilage degeneration in OA by establishing the pathogenic feed-forward loop in chondrocytes, facilitated by the Ca^2+^-cGAS-STING-NF-κB signaling pathway.

TRPA1 ion channel plays a crucial role in promoting joint inflammation in OA (Figure 2). The TRPA1 cation channel is expressed in primary human osteoarthritic chondrocytes, and expression increases in response to inflammatory stimuli, including IL-1β, IL-17, and lipopolysaccharide (LPS) [20]. TRPA1 plays a crucial role in upregulating IL-6 expression in chondrocytes, and knocking out *Trpa1* in chondrocytes suppresses IL-6 expression [35]. Pharmacological inhibition of TRPA1 also reduces the production of MMP-1, MMP-3, MMP-13, and prostaglandin E2 (PGE2) in osteoarthritic chondrocytes and murine cartilage [20]. Additionally, the expression of other IL-6 family cytokines, such as leukemia inhibitory factors (LIF) and IL-11, is reduced in *Trpa1*-deficient chondrocytes. Moreover, TRPA1 antagonists further decrease IL-6 production in both murine and human OA chondrocytes [35].

In summary, TRP channels, including TRPV1, TRPV2, TRPV4, TRPV5, TRPV6, TRPC1, TRPM2, and TRPA1, have been implicated in cartilage homeostasis and in age-related or post-traumatic OA pathogenesis. These TRP channels regulate intracellular Ca^2+^ signaling in chondrocytes, thereby influencing their function and maintaining cartilage integrity. These TRP channels potentially affect mechanotransduction, cell survival, responses to oxidative stress, autophagy, and ECM turnover. Depending on the condition, activation of TRPV1 and TRPV4 can support cartilage integrity under physiological conditions, but when chronically or excessively activated, they promote inflammation, chondrocyte death, matrix degradation, and pain. TRPV2 activation promotes anabolic effects on cartilage by increasing lubricin production and inhibiting chondrocyte hypertrophy and calcification. TRPV5 and TRPM2 are involved in Ca^2+^ dysregulation, oxidative stress, and apoptotic pathways that accelerate cartilage degeneration. In contrast, TRPV6 and TRPC1 typically play protective roles by preserving Ca^2+^ homeostasis, mitigating inflammation, and diminishing chondrocyte senescence in OA. TRPA1 serves as a sensor for inflammatory and oxidative signals, enhancing the production of proinflammatory cytokines and the release of matrix-degrading enzymes, thereby contributing to cartilage damage. Together, these channels integrate mechanical, inflammatory, and metabolic signals to determine cartilage fate and drive OA progression.

## 3. TRP Channels in Synoviocytes and Synovial Inflammation

**Figure 4:**
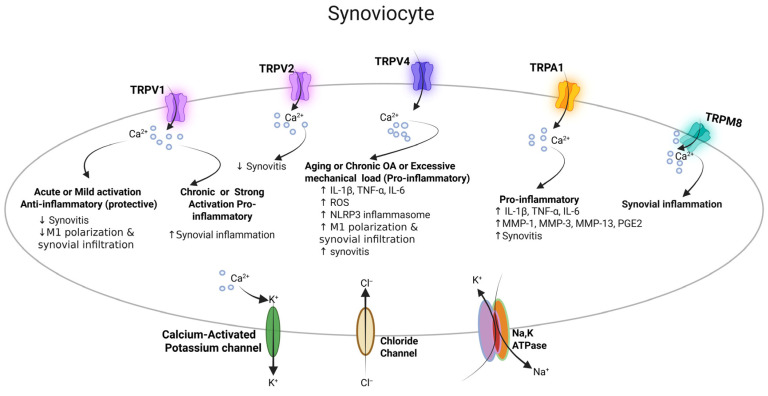
A schematic overview of how TRP channels influence osteoarthritis progression in synoviocytes. Acute activation of TRPV1 induces an anti-inflammatory response in synovial tissue, whereas chronic activation induces a pro-inflammatory response. TRPV4 activation by aging, chronic OA, or excessive loading promotes inflammatory and oxidative stress in the synovium. TRPA1 activation in synoviocytes leads to a pro-inflammatory response and causes synovitis. TRPM8 also causes synovial inflammation.

Synovial membrane inflammation (synovitis) develops in OA and is associated with increases in joint pain and cartilage deterioration. OA models exhibit substantial macrophage infiltration in synovial tissue, with a notable shift toward the pro-inflammatory M1 phenotype [36]. Macrophages within inflamed synovial tissue release proinflammatory cytokines, including TNF-α, IL-1β, and IL-6, chemokines, and enzymes that degrade the cartilage matrix, thereby driving articular cartilage loss and joint inflammation [36]. The synovial inflammatory environment not only damages joint structures but also increases nociceptor sensitivity, resulting in more intense pain and reduced joint function [36]. During OA, macrophage infiltration in synovial and DRG tissues leads to increased expression of TRPA1, TRPV1, and TRPM8 in these areas [36]. Thus, pathological changes in the synovium and peripheral sensory system heighten nociception, partly by modulating the expression of TRP channels, including TRPA1, TRPV1, and TRPM8 in synovial tissues (Figure 4) [36].

Various TRP channels, including TRPA1, TRPV1, TRPV2, TRPV4, and TRPM3, play roles in OA synovial tissue and regulate synovial inflammation and cartilage damage. TRPA1 expression is increased in OA-associated synovial tissue, contributing to cytokine production and synovial inflammation [20,37,38]. Within synovial tissue, TRPA1 is expressed in primary fibroblast-like synoviocytes (FLS), where it mediates the inflammatory response induced by LPS (Figure 4) [37]. LPS increases TRPA1 expression and enhances Ca^2+^ influx in OA FLS. Inhibition of TRPA1 by gene silencing reduces the levels of pro-inflammatory mediators and cartilage-degrading enzymes, including IL-1β, TNF-α, IL-6, MMP-1, and MMP-3. TRPA1 antagonists also lessen joint inflammation and cartilage damage in animal models of arthritis [38,39]. These findings indicate that TRPA1 plays a significant role in OA-related inflammation and joint damage, making it a promising therapeutic target.

TRPV1 has also been implicated in synovial inflammation. Its expression is present in both human and rat synovium and correlates with M1 macrophage infiltration [24]. In a rat OA model, intra-articular injection of the TRPV1 agonist, capsaicin, markedly reduces synovial cell infiltration by macrophages and synovial hyperplasia (Figure 4) [24]. Mechanistically, TRPV1 activation triggers Ca^2+^ influx, leading to phosphorylation of Ca^2+^/calmodulin-dependent protein kinase II (CaMKII) and nuclear translocation of the redox-sensitive Nrf2, thereby suppressing M1 macrophage polarization in the synovium [24]. By suppressing M1 macrophages and dampening synovial inflammation, TRPV1 stimulation diminishes the synovitis component of OA, thus helping to protect against cartilage damage and osteophyte formation.

TRPV2 is expressed in human and rat FLS and functions as a suppressor of OA severity, joint damage, and FLS invasion (Figure 4) [40]. Activation of TRPV2 reduces IL-1β-induced FLS invasion, migration, and MMP-2 and MMP-3 expression, which are key factors in matrix degradation and synovitis formation [40]. Conversely, inhibiting or silencing TRPV2 increases FLS invasiveness, demonstrating that TRPV2 negatively regulates FLS-driven joint destruction [40]. In animal models of OA, activating TRPV2 notably decreases joint inflammation, cartilage erosion, bone damage, and synovial angiogenesis [40]. Mechanistically, TRPV2-mediated Ca^2+^ influx counteracts proinflammatory pathways, suppressing MMP production and inflammatory cytokine release [40]. Therefore, it acts as an anti-inflammatory and anti-invasive regulator in SF.

TNF-α upregulates and activates TRPV1 and TRPV4 channels in human synoviocytes [41]. TNF-α treatment increases Ca^2+^ influx in response to thermal and chemical stimuli, enhancing cellular sensitivity and stimulating the release of inflammatory mediators, such as prostaglandins and cytokines. TNF-α contributes to synovial inflammation not only via cytokine signaling but also by sensitizing TRP channels, linking Ca^2+^ signaling directly to joint inflammation and pain in arthritis [41].

Sun et al. highlighted the role of TRPV4 in linking mechanical and inflammatory stresses to the progression of OA (Figure 4) [11]. Medial meniscus radial transection (MMT) injury upregulates the expression of TRPV4 in rat OA articular cartilage, similar to high levels seen in human OA. TRPV4 expression is associated with an increased influx of M1 macrophages into the OA synovium. TRPV4 activation in a monocyte cell line, RAW264.7, induces Ca^2+^ flux, stimulates mitochondrial ROS production, and activates the nucleotide-binding domain, leucine-rich–containing family, pyrin domain–containing 3 (NLRP3) inflammasome, promoting M1 macrophage polarization and increasing the release of pro-inflammatory cytokines, including IL-1β, IL-6, and TNF-α. Intra-articular injection of a selective TRPV4 inhibitor, HC067047, improved OARSI scores, synovitis, and infiltration of M1 macrophages into the synovium in a rat MMT model. Further mechanistic exploration suggests that TRPV4 inhibition decreases mitochondrial ROS production in macrophages, which in turn suppresses activation of the NLRP3 inflammasome (reducing NLRP3, caspase-1, and cleaved caspase-1 levels), thereby blocking M1 polarization and subsequent production of pro-inflammatory cytokines [11]. Further studies are required to determine whether TRPV4 responds to mechanical stress or to hypoosmotic synovial fluid produced in OA. Thus, TRPV4 inhibition may be a promising therapeutic target for controlling inflammation and limiting post-traumatic OA progression.

Thus, targeting synovial TRP channels, particularly TRPA1, TRPV1, TRPV2, TRPV4 and TRPM8, presents promising therapeutic strategies for OA. Inhibiting TRPA1 may reduce synovial inflammation and cartilage degradation. Modulating TRPA1, TRPV1 and TRPM8 could limit M1 macrophage activity and synovitis. Activating TRPV2 may protect against joint damage by suppressing synovitis. Suppressing TRPV4 could decrease macrophage-driven inflammation and inflammasome activity.

## 4. TRP Channels in Sensory Neurons and OA-Associated Pain

**Figure 5:**
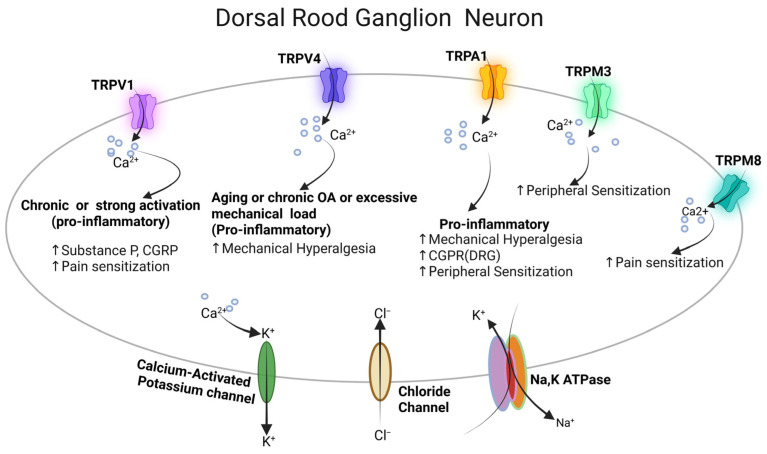
A schematic overview of how TRP channels in DRG neurons influence osteoarthritis related pain and neurogenic inflammation. In sensory neurons, TRPV1, TRPV4, TRPA1, TRPM3 and TRPM8 drive pain sensitivity and neurogenic inflammation.

Findings suggest that synovial inflammation and macrophage-driven immune responses directly promote TRP channel-mediated nociceptive signaling, establishing a mechanistic connection between immune dysregulation, TRPA1, TRPV1, and TRPM8 channel upregulation, and chronic OA pain (Figure 5) [36].

Pain is a key clinical feature of OA, and TRP channels, especially TRPV1, TRPV4, TRPA1, and TRPM3, play a central role in nociceptive pain signaling (Figure 5) [5]. Multiple TRP channels are expressed on sensory neurons within the joint and synovium, where they are upregulated by pro-inflammatory cytokines, including TNF-α, IL-1β, IL-6, and IL-17, and directly influence nociceptive sensory neurons, resulting in peripheral sensitization and hyperalgesia [42]. For instance, TNF-α and IL-1β upregulate TRPV1, which is linked to thermal hyperalgesia, while IL-17 increases TRPV4 expression, which is linked to mechanical hyperalgesia [42]. Neutralizing TNF-α reduces both mechanical and thermal hyperalgesia in the joint, while neutralizing IL-1β lowers thermal hyperalgesia. Additionally, neutralizing IL-6 and IL-17 mainly decreases mechanical hyperalgesia [31].

In OA, the expression and function of TRPV1 in DRG neurons and joint nerve fibers are upregulated, significantly contributing to pain perception (Figure 5) [43,44,45]. Studies in rodent models of OA consistently indicate that TRPV1 sensitization plays a role in both spontaneous pain and mechanically evoked joint pain by mediating pain signals and promoting synovial inflammation [46,47,48,49]. Joint inflammation in OA is also associated with increased levels of pain-inducing molecules such as prostaglandins, bradykinin, and nerve growth factor (NGF), which sensitize nociceptive afferents [50,51]. During inflammation, the heat activation threshold of TRPV1 shifts closer to body temperature, and acidic pH can further sensitize it, allowing normally innocuous stimuli, such as movement or mild warmth, to activate TRPV1, leading to heightened evoked pain sensitivity, including hyperalgesia and allodynia [51]. TRPV1 also contributes to spontaneous OA pain through chronic low pH, inflammatory mediators, and kinase signaling, maintaining its sensitized state. Continuous activation of TRPV1 results in persistent Ca^2+^ influx and heightened nociceptor excitability [52]. Over time, this peripheral activation leads to central sensitization by influencing neurotransmitter release in the dorsal horn, including substance P, calcitonin gene-related peptide (CGRP), and glutamate, thereby increasing nociceptive signaling even in the absence of external stimuli [51,53].

In both animal and human OA, increased TRPV1 expression in DRG and joint afferents has been linked to heightened nociceptor sensitivity and pain transmission, manifesting as mechanical hypersensitivity and weight-bearing asymmetry (Figure 5). Studies involving rodent OA models (MIA and MMT) have demonstrated that TRPV1 plays a pivotal role in both evoked and spontaneous pain. Using capsaicin to desensitize TRPV1 pharmacologically or blocking it with antagonists markedly decreases mechanical and thermal hypersensitivity and persistent pain behaviors, confirming its key role in OA pain mechanisms [47,53,54,55,56]. In the MMT rat model, OA increased TRPV1 expression and pain-related neuropeptides, such as CGRP and Substance P, in the ipsilateral DRG, indicating heightened peripheral sensitization [57]. Treatment with dimethyl fumarate (DMF), which reduces oxidative stress and inflammatory signaling known to sensitize TRP channels, decreases TRPV1-driven Ca^2+^ influx and neuropeptide release, including CGRP and Substance P [57]. These findings suggest that DMF reduces TRPV1 channel–dependent nociceptive signaling and neuroinflammation, positioning it as a potential therapeutic for OA pain and joint degeneration.

Wang et al. also demonstrated that glycine *N*-acyltransferase (GLYAT) deficiency in sensory neurons suppresses OA pain by modulating TRPV1 activity in a MIA OA mouse model [58]. GLYAT, an enzyme in acylglycine metabolism, regulates neuronal excitability and pain sensitivity by modulating TRPV1 [58]. Overexpression of GLYAT causes mechanical and thermal hyperalgesia. Loss of GLYAT reduced acylglycine-mediated activation of TRPV1, thereby diminishing Ca^2+^ influx and neuronal hyperexcitability in DRG neurons. As a result, GLYAT-deficient mice exhibited reduced pain behaviors and lower expression of pain-associated neuropeptides in OA models. GLYAT overexpression disrupts redox balance and increases ROS levels in DRG neurons, thereby upregulating TRPV1 [58]. Additionally, blocking ROS clearance or TRPV1 in GLYAT-overexpressing mice increased mechanical and thermal withdrawal thresholds, indicating reduced pain [58]. Thus, TRPV1 is a key downstream effector of GLYAT-dependent signaling in sensory neurons in OA.

NGF signaling through tropomyosin receptor kinase A (TrkA) receptors also enhances peripheral hyperalgesia by increasing TRPV1-positive sensory nerve growth in the synovium during OA development [59]. Rats with OA show higher levels of TRPV1, the pan-neuronal marker PGP9.5, and inflammatory mediators like IL-1β in synovial tissue, as well as elevated TRPV1, PGP9.5, and S100 proteins in the DRG [59]. This correlates with the sprouting of TRPV1-labeled sensory nerve fibers, which intensifies nociceptive signaling. Blocking TrkA decreases TRPV1-positive innervation and reduces mechanical and cold hyperalgesia, emphasizing the role of the NGF/TrkA-TRPV1 axis in connecting synovial inflammation to TRP channel-mediated pain sensitization in OA [59].

The TRPV4 is another thermosensor that recognizes non-painful warmth. In sensory neurons, TRPV4 is expressed within the DRG, where it influences nociceptive signaling (Figure 5) [60]. The expression of TRPA1 and TRPV4 is upregulated in the DRG and synovial tissues in rat MIA-induced OA joints compared to control joints [61]. Behavioral testing in OA mice shows a significant reduction in paw withdrawal threshold, indicating heightened mechanical hyperalgesia [61]. Pharmacological blockade of TRPA1 or TRPV4 effectively reverses mechanical hypersensitivity, demonstrating that both channels contribute to OA-associated pain [61]. Mechanistically, mechanical stress and inflammatory mediators can activate TRPA1 and TRPV4 channels on peripheral sensory neurons, leading to an influx of Ca^2+^ ions, increased neuronal hyperexcitability, and the release of neuropeptides. This process worsens local inflammation and pain signaling. TRPA1 and TRPV4 synergistically sustain chronic mechanical hyperalgesia and inflammation in osteoarthritic joints, underscoring their roles in pain mediation via nociceptive sensitization and neuroinflammation.

TRPA1 is also a key contributor to OA-associated pain, and it is expressed in nociceptive neurons and synovial tissue, where it mediates mechanical hypersensitivity and inflammatory pain in a rat OA model (Figure 5) [62]. TRPA1 activity directly regulates CGRP expression in DRG neurons, linking TRPA1 signaling to peripheral sensitization and pain transmission. Blocking or deleting TRPA1 reduced pain without affecting cartilage degeneration, indicating that TRPA1 primarily modulates pain pathways rather than joint structure [62]. In vivo targeting of Bromodomain-containing protein 4 (BRD4)-mediated macrophage polarization not only decreases M1 macrophage buildup and synovial inflammation but also reduces TRPA1 expression, alleviating joint pain and preventing cartilage degradation [63]. These findings highlight a neuroimmune-TRP axis in OA, in which immune-driven synovial inflammation controls TRP channel expression in sensory neurons, linking macrophage activity to TRP-dependent pain signaling. Another study demonstrated the role of TRPA1 in chronic arthritis using *Trpa1*-deficient mice [64]. It was found that loss of *Trpa1* markedly reduced pain behaviors, such as mechanical and thermal hypersensitivity, in models of chronic joint inflammation, indicating that TRPA1 contributes directly to the sensory perception of arthritis pain [64]. Another study using the MIA model showed that *Trpa1*-deficient mice have reduced mechanical hyperalgesia [39]. Additionally, proinflammatory mediators, including COX-2, IL-6, and the neuropeptide Substance P, are decreased in *Trpa1*-deficient mice [39]. TRPA1 inhibition reduces CGRP expression, suppressing neuronal excitability and neuropeptide release [65]. These findings suggest that TRPA1 contributes to both inflammatory responses and nociceptive signaling in OA. The above studies also suggested that TRPA1 mediates nociceptive signaling in inflamed joints, underscoring its role as a key regulator of chronic joint pain, linking joint inflammation to sensory perception. Thus, TRPA1 inhibition could be a potential therapeutic for pain management in chronic inflammatory joint diseases.

TRPM3 is a nociceptor channel that contributes to the detection of noxious thermal stimuli and may have a role in OA pain [66]. In peripheral sensory neurons, particularly the trigeminal ganglion and DRG neurons, TRPM3 activation by high temperatures induces cation influx and neuronal depolarization, triggering action potentials and pain signals (Figure 5) [66,67]. Inhibiting or knocking out TRPM3 significantly reduces heat-induced neuronal activity and pain behaviors, confirming its importance in heat-related nociception [66]. Although most studies focus on heat pain, the presence of TRPM3 in joint-innervating sensory neurons suggests it may also contribute to OA pain by mediating thermal hyperalgesia and peripheral sensitization. Thus, TRPV1, TRPV4, TRPA1, TRPM3 and TRPM8 highlight their significant contributions to sensory pain signaling in OA.

## 5. Signaling in the Osteoarthritis Pathogenesis

In OA, the NF-κB, Mitogen-activated protein kinase (MAPK), Janus kinase/signal transducer and activator of transcription (JAK/STAT), and phosphoinositide 3-kinase/protein kinase B/mammalian target of rapamycin (PI3K/AKT/mTOR) signaling pathways primarily influence inflammation, cartilage degradation, and bone remodeling [68,69]. The NF-κB signaling pathway is activated by mechanical stress, cytokines (TNF-α, IL-1β, IL-6), pattern-recognition receptors, and Toll-like receptor signals. NF-κB induces the transcription of genes associated with catabolism and inflammatory responses, via the phosphorylation of the inhibitor of nuclear factor κB (IκB) by IκB kinase (IKK), releasing NF-κB dimers (p65/p50), which translocate to the nucleus and increase the production of MMPs (MMP1, MMP9, MMP13) and ADAMTS5, thereby promoting degradation of the ECM [69]. NF-κB also induces the expression of pro-inflammatory mediators, such as COX-2, NOS, and PGE2, thereby driving cartilage inflammation and chondrocyte apoptosis [69,70,71]. This sequence establishes a positive feedback loop that perpetuates joint inflammation and tissue damage [72]. TRPV4 activation controls Ca^2+^ influx, activating the CaMKK/AMPK pathway that suppresses NF-κB, reducing pro-inflammatory cytokines and matrix-degrading enzymes in chondrocytes [73].This mechanism helps prevent cartilage damage and maintains chondrocyte health, thereby protecting cartilage via the CaMKK/AMPK/NF-κB axis.

TRPV4 also has anti-inflammatory effects, and its activation in chondrocytes helps dampen inflammation and preserve cartilage [73]. Pharmacological activation of TRPV4 with the agonist GSK101 reverses IL-1β–induced increases in *MMP13* and restores levels of *Acan* and SRY-box transcription factor 9 (*Sox9*) mRNA in both bovine and human articular chondrocytes [73]. Furthermore, TRPV4 activation reduces IL-1β–induced sulfated glycosaminoglycan (sGAG) release in bovine cartilage explants and preserves Safranin O staining, thereby protecting cartilage tissue [73]. Mechanistically, TRPV4 activation increases AMPK phosphorylation and suppresses NF-κB phosphorylation in IL-1β-stimulated chondrocytes. Inhibition of AMPK with Compound C or CaMKK with STO-609 abolishes the suppressive effect of GSK101 on NF-κB activation and MMP-13 expression [73]. Collectively, TRPV4 activation maintains chondrocytes in a less catabolic state by activating Ca^2+^-dependent CaMKKβ/AMPK signaling, which promotes anabolic gene expression (e.g., *Sox9*, *Acan*), suppresses catabolic enzymes (e.g., MMP-13), and inhibits NF-κB-driven inflammation, thereby preserving ECM homeostasis [73]. While TRPV4-mediated Ca^2+^ signaling activates CaMKKβ/AMPK, which suppresses NF-κB–driven inflammation and promotes chondrocyte protection [73], excessive mechanical stress can abnormally activate TRPV4, contributing to damaging effects on cartilage.

In OA, pro-inflammatory cytokines such as IL-1β and TNF-α bind to their receptors and activate signaling cascades that stimulate the IKK complex (Figure 3). IKK phosphorylates IκBα, leading to its ubiquitination and degradation. This process releases NF-κB dimers (p65/p50), which move into the nucleus and bind to target gene promoters. The resulting transcriptional increase in pro-inflammatory and catabolic mediators, including IL-6, COX-2, iNOS, MMPs, and ADAMTS5, exacerbates inflammation and cartilage breakdown in OA.

PIEZO1 coordinates the mechanical or pharmacological activation of TRPV4, which promotes anti-inflammatory and anabolic responses in chondrocytes (Figure 3). This activation increases Ca^2+^ influx, which in turn activates CaMKK. CaMKK then stimulates AMPK, a key regulator of cellular energy and inflammation. Activated AMPK suppresses NF-κB signaling, reducing the transcription of pro-inflammatory cytokines such as IL-1β and TNF-α, as well as matrix-degrading enzymes such as MMPs. Simultaneously, AMPK activation increases the expression of anabolic genes and cartilage-protective factors, including *Sox9* and *Acan*, thereby supporting ECM synthesis and cartilage integrity (Figure 3). Thus, TRPV4-mediated Ca^2+^ signaling acts as a key counter-regulatory mechanism in chondrocytes. It connects mechanical or agonist-induced stimuli to anti-inflammatory and anabolic pathways, opposing the catabolic effects of NF-κB activation and helping protect cartilage from osteoarthritis-related damage.

The MAPK signaling pathway is activated by cytokines, growth factors, oxidative stress, and by MAPK cascades, which transmit signals through phosphorylation to regulate inflammation, catabolism, and ECM breakdown. Inflammatory stimuli, such as IL-1, activate MAPKs, resulting in increased MMP production and cartilage damage [74]. Notably, c-Jun *N*-terminal kinase (JNK) and p38 MAPK contribute to chondrocyte apoptosis [75], while ERK1/2 and p38 are involved in bone and cartilage crosstalk during OA progression [76,77]. Inflammatory stimuli often trigger Ca^2+^ influx via TRP channels, thereby connecting them to the regulation of the MAPK signaling pathway. TRPV5 upregulation in osteoarthritic chondrocytes triggers CaMKII phosphorylation and downstream MAPK signaling, resulting in chondrocyte apoptosis [78]. Similarly, TRPM8 activation enhances p-ERK and NF-κB signaling to amplify inflammation, and TRPA1-mediated mechanical stimulation promotes ERK1/2 and p38 phosphorylation in DRG neurons [79,80].

During the development of OA, pro-inflammatory cytokines such as IL-1β, IL-6, and TNF-α bind to cell membrane receptors, thereby activating JAK kinases [68]. These kinases subsequently phosphorylate STAT transcription factors, which then dimerize and regulate genes that promote synovial inflammation and cartilage degeneration [81]. In chondrocytes, activation of the JAK/STAT3 pathway by IL-1β or IL-6 leads to an upregulation of MMPs, driving cartilage matrix degradation and exacerbating cartilage damage [82,83]. Furthermore, this pathway enhances synovial inflammation and facilitates OA progression by increasing IL-6 production. Additionally, this pathway induces pathological angiogenesis in the subchondral bone via Receptor Activator of Nuclear Factor kappa-B Ligand (RANKL), Elongation protein 2 (ELP2) factors, and Hypoxia-Inducible Factor Alpha/Vascular Endothelial Growth Factor (HIF-α/VEGF) signaling, collectively contributing to worsening OA progression [84,85,86,87].

The PI3K/AKT/mTOR pathway plays a central role in OA by regulating chondrocyte survival, inflammation, and cartilage matrix homeostasis. Pro-inflammatory cytokines, such as IL-1β, activate PI3K, leading to phosphorylation of AKT and mTOR, which, in turn, increase the production of inflammatory mediators, including IL-1β and nitric oxide (NO), and MMPs by chondrocytes, which promotes cartilage matrix degradation. Moreover, the PI3K/AKT pathway interacts with NF-κB, amplifying inflammatory responses and establishing a feedback loop that exacerbates synovial inflammation and OA progression. Importantly, TRP channels such as TRPV4 influence these signaling cascades; TRPV4-mediated Ca^2+^ influx modulates PI3K/AKT and MAPK/ERK pathways, controlling chondrocyte survival and death, and driving fibroblast-to-myofibroblast differentiation [88,89,90]. Together, these interactions illustrate how mechanosensitive ion channels integrate inflammatory and mechanical cues to regulate joint degeneration in OA.

In patients with OA, serum and synovial fluid analyses revealed elevated levels of proinflammatory cytokines and chemokines, including IL-17A, C-X-C motif chemokine ligand (CXCL)8, CXCL9, and CXCL11 [91]. Among these, CXCL8 and CXCL11 exerted strong pathogenic effects on chondrocytes by promoting apoptosis and inhibiting proliferation, probably through activation of the JAK/STAT, NF-κB, and JNK-MAPK signaling pathways, thereby further enhancing the expression of other inflammatory mediators [91]. The resulting inflammatory milieu not only accelerates cartilage degradation but also contributes to peripheral sensitization by upregulating nociceptive TRP channels such as TRPV1 and TRPA1 in joint-innervating sensory neurons [36,57,59]. Thus, CXCL8 and CXCL11 act as upstream modulators linking synovial inflammation to TRP channel–mediated nociceptive signaling, amplifying pain and tissue degeneration in OA.

## 6. Integration of Key TRP Channels in OA Pathophysiology

The overall evidence shows that TRP channels play a key role in various aspects of OA, including mechanotransduction, chondrocyte survival and catabolism, synovial inflammation, and nociceptive signaling. TRP channels play central and context-dependent roles in OA pathophysiology by integrating mechanical, inflammatory, and neurogenic signals across joint tissues. TRPV1 exhibits dual effects depending on the intensity and duration of activation. Acute or mild TRPV1 activation exerts a protective effect against inflammation by lowering lipid peroxidation, iron accumulation, oxidative stress, and ferroptosis in articular chondrocytes (Figure 2). It also reduces M1 macrophage polarization and immune cell infiltration in the synovium, thereby reducing synovitis. In contrast, chronic or excessive TRPV1 activation promotes inflammation, characterized by increased IL-1β, TNF-α, and IL-6 production, enhanced cartilage degradation in chondrocytes, heightened synovial inflammation, and increased release of substance P and CGRP from joint-innervating sensory neurons, ultimately amplifying pain sensitization (Figure 4 and Figure 5). TRPM3 also increases pain sensitization in sensory nerves (Figure 5). TRPV2 plays a chondroprotective role by increasing lubricin production and suppressing joint inflammation, cartilage erosion, and bone damage (Figure 2 and Figure 4).

TRPA1, TRPV1, and TRPM8 have been seen expressed in both synoviocytes and DRG and are directly related to synovial inflammation and pain sensitization during OA (Figure 4 and Figure 5).

TRPV4 plays different roles depending on the amount of mechanical load. Under normal conditions, TRPV4 signaling helps protect cartilage by reducing MMP-13 expression and NF-κB-driven inflammation, while increasing anabolic markers such as ACAN and SOX9 (Figure 2). These effects help maintain cartilage health. However, as people age, develop OA, or experience excessive mechanical stress, continued TRPV4 activation may become harmful. It can lead to chondrocyte apoptosis or pyroptosis, cartilage damage, synovial inflammation, M1 macrophage polarization, and higher levels of IL-1β, TNF-α, IL-6, ROS, and NLRP3 inflammasome activity. In sensory neurons, TRPV4 is involved in mechanical hyperalgesia.

TRPV5 activation in chondrocytes promotes autophagy and increases apoptosis and cartilage matrix degradation, whereas TRPV6 exhibits a chondroprotective effect and reduces inflammation, and promotes anabolic activity of cartilage (Figure 2). TRPA1 mainly acts as a pro-inflammatory factor, causing cartilage breakdown in chondrocytes, increasing synoviocyte production of IL-1β, TNF-α, IL-6, MMP-1, MMP-3, MMP-13, PGE2, and synovitis, and raising mechanical pain sensitivity, CGRP release, and nerve sensitization in DRG sensory neurons (Figure 2, Figure 4 and Figure 5). TRPC1 supports chondrocyte health by preserving type II collagen and ACAN, reducing cartilage damage in OA, and limiting chondrocyte dedifferentiation and senescence (Figure 2). In contrast, TRPM2 drives inflammation and catabolic activity by increasing pro-inflammatory cytokines (IL-1β, TNF-α, IL-6), catabolic enzymes (COX-2, iNOS, MMP-13, ADAMTS5), ROS production, chondrocyte apoptosis, ECM degradation, and loss of *Col2A1* and *Acan* expression, as well as exacerbating synovitis (Figure 2).

As highlighted by Mobasheri et al., TRP channels do not work alone, but are part of a larger network of ion channels in chondrocytes, often referred to as the “channelome” [92]. In addition to TRP channels, other ion transport systems, including potassium (K^+^) channels, chloride (Cl^−^) channels, and the Na^+^/K^+^-ATPase pumps, are also expressed and functionally active in chondrocytes, synoviocytes, and DRG sensory neurons (Figure 2, Figure 4 and Figure 5), where they contribute to membrane potential regulation, ionic homeostasis, mechanotransduction, inflammatory signaling, and pain transmission.

Mechanically sensitive TRP channels, such as TRPV4 and TRPV5/6, respond to abnormal cartilage loading, leading to either protective or apoptotic Ca^2+^ signals. Meanwhile, nociceptive channels, such as TRPV1 and TRPA1, translate synovial inflammation and cartilage injury into pain. Importantly, therapeutic strategies targeting TRP channels can be designed to focus on preserving cartilage, reducing synovial inflammation, or alleviating pain, depending on the specific channel and whether the treatment involves activation or inhibition.

Recent studies also reveal intricate interactions between TRP channels and other signaling pathways. For example, in osteoarthritic chondrocytes, the TRPV4-glycogen synthase kinase 3 beta (GSK3β) signaling pathway is dysfunctional, impairing the cells’ ability to detect and respond to changes in ECM viscoelasticity [93]. Typically, TRPV4 channels act as mechanosensors that detect ECM stiffness and modulate downstream pathways, such as GSK3β, thereby regulating cytoskeletal dynamics and matrix production. In OA, this mechanotransduction process is disrupted, leading to defective cytoskeletal remodeling, reduced ECM synthesis, and compromised cartilage homeostasis, all of which contribute to disease progression. Similarly, in osteoarthritic chondrocytes, TRPV5-mediated Ca^2+^ influx inhibits autophagy and promotes apoptosis via CaMKII-dependent activation of MAPK and Akt/mTOR, leading to cartilage degeneration [78]. These mechanistic insights indicate that TRP channels are not just sensors but also active regulators of OA progression.

## 7. OA Pathophysiology: Current Perspectives on TRP Channels

Recent advances in OA research increasingly recognize the critical role of various ion channels in articular chondrocytes, which regulate joint homeostasis, nociception, and disease progression. Many channel proteins are multifunctional, serving as ion channels, receptors, and signaling hubs. Mobasheri and colleagues have given the term “Chondrocyte Channelome” as a key conceptual framework for understanding OA pathophysiology [92,94]. They described various ion channels in articular chondrocytes, such as mechanosensitive, voltage-gated, and ligand-gated channels. Disruptions in channel activity impact Ca^2+^ signaling, ECM synthesis, and chondrocyte viability. This shifted the view of OA from merely mechanical cartilage erosion to a disease partly caused by abnormal ion-channel signaling. Subsequently, this perspective was broadened, as it was found that ion channels such as TRP, acid-sensing ion channels (ASIC), PIEZO, and voltage-gated Ca^2+^ channels are key mediators of inflammatory hyperexcitability and nociception in osteoarthritis and related musculoskeletal disorders [95].

TRP channels, notably TRPV1, TRPA1, and TRPV4, play vital roles in inflammation and pain associated with OA. They respond to mechanical overload, acidic pH, ROS, and inflammatory signals, adapting to the OA microenvironment. TRPV1 and TRPA1 are widely expressed in joint tissues, including chondrocytes, synoviocytes, osteocytes, and sensory neurons, and become more sensitive during inflammation. When activated in chondrocytes and synoviocytes, they induce Ca^2+^ influx, triggering cytokine release (e.g., IL-6), the production of matrix-degrading enzymes, and increased nociceptor activity. In sensory neurons, these channels lower activation thresholds, thereby promoting peripheral sensitization and chronic OA pain, whereas their upregulation in DRG enhances central pain pathways. Moreover, TRPA1 is involved in cartilage degeneration and acute joint inflammation, whereas TRPV1 affects macrophage polarization, potentially reducing synovitis upon activation. Genetic evidence linking TRPV1 to OA pain, along with clinical trials targeting these channels, underscores their promise as therapeutic targets for OA pain management.

Furthermore, there is a crucial interplay between inflammatory mediators and mechanotransduction, which is mediated by ion channels in articular chondrocytes [96]. Chondrocytes rely on a complex “channelome” that includes TRP and PIEZO mechanosensitive channels, voltage-gated Ca^2+^ channels, and various K^+^ and Cl^−^ conductance to process mechanical and inflammatory stimuli. In OA, pro-inflammatory cytokines (such as IL-1β and TNF-α), altered osmolarity, acidity, and abnormal loading disrupt normal channel function, leading to aberrant Ca^2+^ influx, membrane depolarization, and impaired mechanotransduction. These changes increase inflammatory and catabolic gene expression, enhance chondrocyte sensitivity to mechanical load, and reduce matrix synthesis, thereby promoting cartilage degeneration. Thus, mechanotransduction and inflammation are not separate processes but rather integrated pathways, largely controlled by ion channels.

Additionally, TRPV1, a nociceptor activated by heat, protons, and inflammatory mediators, is abundantly expressed in nociceptive neurons innervating the knee joints [97]. In OA, its expression often increases in synovium, bone, and cartilage, indicating pathological sensitization. Injecting TRPV1 agonists, such as capsaicin or Resiniferatoxin, directly into the joint can desensitize pain-sensing nerve fibers, providing long-lasting relief without systemic side effects, such as hyperthermia, observed with systemic TRPV1 antagonists. This shifts the understanding of OA pain from a focus on damage or inflammation to an active, modifiable signaling process, making TRP-channel modulation a possible, joint-specific strategy for pain reduction and OA progression.

Peripheral analgesia targets pain at its source, aligning with TRP channels’ role in OA. TRPV1, TRPA1, and TRPV4 act as joint sensors for inflammation, mechanical stress, and oxidative damage. In OA, these channels become hyperactive, causing excess Ca^2+^ influx into chondrocytes and synoviocytes and sensitizing peripheral nociceptors that innervate the joint [98]. This creates a self-reinforcing cycle of inflammation, matrix breakdown, and increased pain perception. Thus, these channels promote both inflammatory signaling within chondrocytes and the sensitization of peripheral nociceptors. Targeting these channels provides a precise way to block nociceptive signaling before it reaches the central nervous system, providing effective, localized pain relief with few side effects. Evidence supports TRP channels as promising targets for mechanism-based OA pain treatment.

Although cytokine-driven signaling in chondrocytes and synoviocytes is a key aspect of OA pathophysiology, a more comprehensive understanding of joint dysfunction also requires consideration of changes in the production and release of essential lubricants, particularly proteoglycan 4 (PRG4). Lubricin, encoded by PRG4, is traditionally known for its role in lubrication, maintaining joint homeostasis by minimizing friction and preventing cartilage wear, and is now also seen as a crucial regulator of tissue health and inflammation. SF and superficial zone chondrocytes mainly produce it [99]. Disruption of lubricin production or secretion is a hallmark of OA progression and contributes to cartilage friction, surface fibrillation, and synovial inflammation.

Rhee et al. established that lubricin is a critical secreted glycoprotein that preserves joint integrity by protecting articular cartilage surfaces and restraining synovial cell overgrowth [100]. Loss of lubricin results in increased cartilage friction, damage surfaces, and causes abnormal synovial cell growth, leading to joint dysfunction [100]. Lubricin functions as a boundary lubricant and anti-adhesive agent, preventing direct contact between cartilage surfaces and reducing abnormal synovial tissue growth [100]. These findings support the view that lubricin is crucial for maintaining joint surface stability and preventing degenerative changes associated with arthropathy.

TRPV2 has been identified as an essential regulator of articular cartilage homeostasis through its control of lubricin (PRG4) expression [21]. In mouse articular cartilage, TRPV2-driven Ca^2+^ signaling stimulates lubricin production in chondrocytes, aiding boundary lubrication and maintaining the non-hypertrophic cartilage phenotype. When TRPV2 activity is impaired, lubricin levels decrease, and chondrocytes tend to become hypertrophic, which can cause abnormal endochondral ossification in cartilage. These results identify TRPV2 as a mechanosensitive upstream regulator that links Ca^2+^ signaling to lubricin expression and the suppression of pathological cartilage ossification, suggesting its importance in osteoarthritis pathogenesis.

Das et al. provide evidence that PRG4 can modulate inflammatory responses and immune cell activity and protect cartilage from shear-induced damage [101]. Synovial fibroblasts are a key source of PRG4, and their dysregulation in OA leads to reduced lubrication and a more pro-inflammatory cytokine secretion, accelerating joint degeneration. Notably, these fibroblasts express several TRP channels, including TRPV1, TRPA1, and TRPV4, which respond to inflammatory mediators, mechanical stress, and oxidative signals typical of the OA microenvironment. TRP-mediated Ca^2+^ influx in SF can influence gene expression programs that control PRG4 production, cytokine release, and matrix remodeling, positioning TRP channels as upstream regulators of both lubrication and inflammation. Connecting PRG4 biology with TRP channel signaling provides a broader, more precise view of OA progression, showing that TRP channels are involved not just in pain sensation and signaling degeneration but also in disrupting boundary lubrication systems essential for joint health.

## 8. Conclusions

TRP channels are increasingly recognized as vital mediators in the intricate pathophysiology of OA. These ion channels, expressed in chondrocytes, synoviocytes, and DRG neurons, respond to mechanical, thermal, chemical, and osmotic stimuli, which are prevalent within the osteoarthritic joint environment. Their activation influences Ca^2+^ signaling, thereby regulating various cellular processes, including cell proliferation, autophagy, apoptosis, oxidative stress, ECM degradation, chondroprotection, release of inflammatory mediators, synovial macrophage polarization and infiltration, and pain sensitivity. Dysregulation of TRP channel activity contributes to cartilage degradation, synovial inflammation, and altered nociceptive signaling, all of which are key features of OA progression. Some TRP channels serve a homeostatic role that is cartilage protective, but drive cartilage catabolism during excessive stress, strain, or channel activation. Modulating their activity could control detrimental effects and slow the progression of OA.

Thus, because of their central role in connecting mechanical stress, inflammation, and pain, targeting TRP channels locally in the joint may have a role in OA management in the future. However, translation of TRP channel agonists and antagonists from preclinical models to human conditions has revealed untoward side-effect profiles, including skin cancers and increased body temperature. Future research focusing on cell-specific targeting and AI-influenced drug design may allow more precise targeting of TRP channels to limit side effects.

## Data Availability

No new data were created or analyzed in this study.

## Abbreviations

Acan	Aggrecan
ADAMTS	A disintegrin and metalloproteinase with thrombospondin motifs
AKT	Protein kinase B
AMP	Adenosine monophosphate
AMPK	AMP-Activated protein kinase
ASIC	Acid-sensing ion channels
bFGF	Basic fibroblast growth factor
BRD4	Bromodomain-containing protein 4
Ca^2+^	Calcium
CALCA	Calcitonin-related polypeptide alpha
CaMKII	Ca^2+^/calmodulin-dependent protein kinase II
CaMKK	Ca^2+^/calmodulin-dependent protein kinase kinase
cGAS	Cyclic GMP–AMP synthase
CGRP	Calcitonin gene–related peptide
Cl^−^	Chloride
Col10a1	Collagen type X alpha 1 chain
Col2a1	Collagen type II alpha 1
COX-2	Cyclooxygenase-2
CXCL	C-X-C motif chemokine ligand
DMF	Dimethyl fumarate
DMM	Destabilized medial meniscus
DRG	Dorsal root ganglia
ECM	Extracellular matrix
ELP2	Elongation protein 2
ER	Endoplasmic reticulum
ERK	Extracellular signal-regulated kinase
FLS	Fibroblast-like synoviocytes
GLYAT	Glycine *N*-acyltransferase
GPX4	Glutathione Peroxidase 4
GSK3β	Glycogen Synthase Kinase 3 beta
HIF-α	Hypoxia-Inducible Factor Alpha
IKK	IκB kinase
IκB	Inhibitor of nuclear factor κB
JAK/STAT	Janus kinase/signal transducer and activator of transcription
JNK	c-Jun N-terminal kinase
K^+^	Potassium
LC3B	Light chain 3B
LIF	Leukemia inhibitory factors
LPS	Lipopolysaccharide
MAPK	Mitogen-activated protein kinase
MIA	Monosodium iodoacetate
MMPs	Metalloproteinases
MMT	Medial meniscus radial transection
mTOR	Mammalian target of rapamycin
NF-κB	Nuclear factor kappa-light-chain-enhancer of activated B cells
NGF	Nerve growth factor
NLRP3	Nucleotide-binding domain, leucine-rich–containing family, pyrin domain–containing 3
NO	Nitric oxide
Nrf2	Nuclear factor erythroid 2-related factor 2
OA	Osteoarthritis
OARSI	Osteoarthritis research society international
p16INK4a	p16 inhibitor of kinase 4a
PGE2	Prostaglandin E2
PI3K	Phosphoinositide 3-kinase
PIEZO1	Piezo-type mechanosensitive ion channel component 1
PRG4	Proteoglycan 4
RANKL	Receptor Activator of Nuclear Factor kappa-B Ligand
ROS	Reactive oxygen species
Runx2	Runt-related transcription factor 2
SA-β-gal	Senescence-associated β-galactosidase
SF	Synovial fibroblasts
sGAG	Sulfated glycosaminoglycan
SLC7A11	Solute carrier family 7 member 11
Sox9	SRY-box transcription factor 9
STING	Stimulator of interferon genes
TAC1	Tachykinin Precursor 1
TrkA	Tropomyosin receptor kinase A
TRP	Transient receptor potential
TRPA	TRP Ankyrin
TRPC	TRP Canonical
TRPML	TRP Mucolipin
TRPM	TRP Melastatin
TRPN	TRP No-mechano-potential
TRPP	TRP Polycystin
TRPV	TRP Vanilloid
VEGF	Vascular Endothelial Growth Factor
